# Progressive Hemiparesis Revealing an Atypical Large-Vessel Vasculitis: A Case Report Tackling a Diagnostic Overlap

**DOI:** 10.1159/crn/abyag001

**Published:** 2026-08-10

**Authors:** Rami Abdul Malak, Idriss Youcef Zoubeïdi, Julien Vanderhulst, Anissa Ourtani

**Affiliations:** a Department of Neurology, Centre Hospitalier Universitaire (CHU) Brugmann, Brussels, Belgium; b Department of Internal Medicine, CHU Brugmann, Brussels, Belgium; c Department of Neurology, Universitair Ziekenhuis (UZ) Brussel, Brussels, Belgium; d Neuroprotection and Neuromodulation (NEUR) Research Group, Center for Neurosciences (C4N), Vrije Universiteit Brussel (VUB), Brussels, Belgium

**Keywords:** Case report, Large-vessel vasculitis, Stroke

## Abstract

**Introduction:**

Large-vessel vasculitis (LVV) is a rare cause of ischemic stroke and may pose significant diagnostic challenges, particularly when classical systemic or inflammatory features are absent. Distinguishing between giant cell arteritis and Takayasu arteritis can be especially difficult in older patients with overlapping clinical and radiological findings.

**Case Presentation:**

We report the case of a woman in her 60s presenting with progressive left-sided weakness and multifocal ischemic strokes. Vascular examination revealed marked inter-arm blood pressure asymmetry, absent right radial pulse, and diffuse arterial bruits. Imaging demonstrated extensive circumferential thickening and stenosis of the aorta and its major branches. Extensive investigations excluded cardioembolic, thrombotic, malignant, and infectious causes. Inflammatory markers were normal, whole-body ^18^F-fluorodeoxyglucose positron emission tomography-computed tomography showed no hypermetabolic activity, and temporal artery biopsy was nondiagnostic. The clinical and imaging phenotype was consistent with LVV-spectrum disease, although the patient did not fulfill ACR/EULAR classification criteria for either giant cell arteritis or Takayasu arteritis. She was treated with corticosteroids and methotrexate, with stabilization of neurological deficits.

**Conclusion:**

This case illustrates the limitations of existing classification frameworks when confronted with borderline or overlapping LVV phenotypes, particularly in elderly patients with atypical presentations, and underscores the importance of clinical judgment when evaluating atypical stroke presentations.

## Introduction

Around 5% of all strokes can be attributed to uncommon causes, including systemic vasculitides [1]. Giant cell arteritis (GCA) is considered the most frequent form of vasculitis in adulthood, and its propensity to occur in patients after the 5th decade is considered a key criterion for its diagnosis [2]. Another large-vessel vasculitis (LVV) is Takayasu’s arteritis (TAK), typically affecting women between 20 and 40 years of age, although later onset or delayed diagnosis has been reported [3]. The clinical and radiological overlap between these two entities represents a significant diagnostic and therapeutic challenge, especially in older patients [4].

This case report adheres to the CARE guidelines for case reporting, and the CARE Checklist is included as online supplementary material (for all online suppl. material, see https://doi.org/10.1159/crn/abyag001).

## Case Report/Case Presentation

A woman in her 60s presented to the emergency department with left upper limb weakness. She reported progressive weakness of the left arm that had begun 5 days prior to admission. She also described a similar episode affecting her left leg 5 months earlier, which had remained stable thereafter. Her medical history was significant for hypercholesterolemia, gastritis, and depression. She smoked one pack of cigarettes per day.

Blood pressure was 154/98 mmHg in the left arm and 80/50 mmHg in the right arm. The remaining vital signs were within normal range. Physical examination revealed diffuse vascular bruits (carotid, subclavian, and abdominal aorta) and an absent right radial pulse. No livedo, purpura, petechiae, or Raynaud phenomenon was observed. Neurological examination demonstrated left arm and left leg motor drift, left leg spasticity with a spastic gait, and left hemispatial neglect. The National Institutes of Health Stroke Score (NIHSS) was 3. The rest of the examination was unremarkable, with preserved orientation, intact cranial nerves, normal sensory examination, and flexor plantar responses. She denied headaches, scalp tenderness, visual disturbances, jaw or limb claudication, morning stiffness, and proximal muscle pain.

Erythrocyte sedimentation rate and C-reactive protein (CRP) levels were within normal range. Computed tomography (CT) of the head performed in the emergency setting revealed multiple subacute ischemic lesions in the right hemisphere. CT angiography (CTA) initially suggested the presence of a mural thrombus within the aortic arch (shown in Fig. 1), extending into the right brachiocephalic trunk, left subclavian artery, and both common carotid arteries, with distal reconstitution beyond the carotid bifurcation (shown in Fig. 2, 3). Given the initial concern for an embolic source based on multifocal infarcts and the suspected mural thrombus, intravenous heparin was initiated pending further diagnostic clarification.

**Fig. 1.**
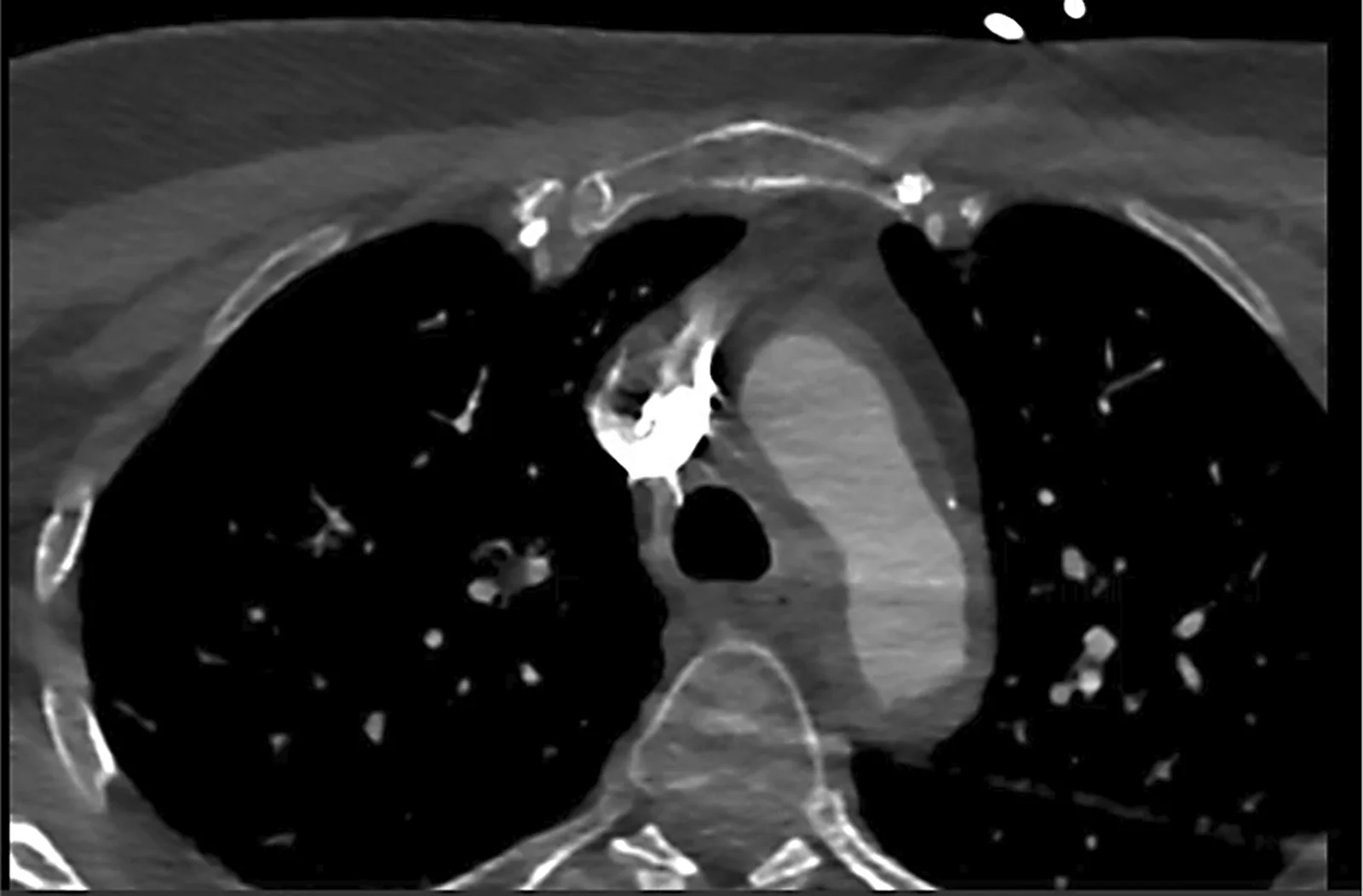
Axial CTA of the aortic arch showing diffuse circumferential wall thickening with associated luminal narrowing.

**Fig. 2.**
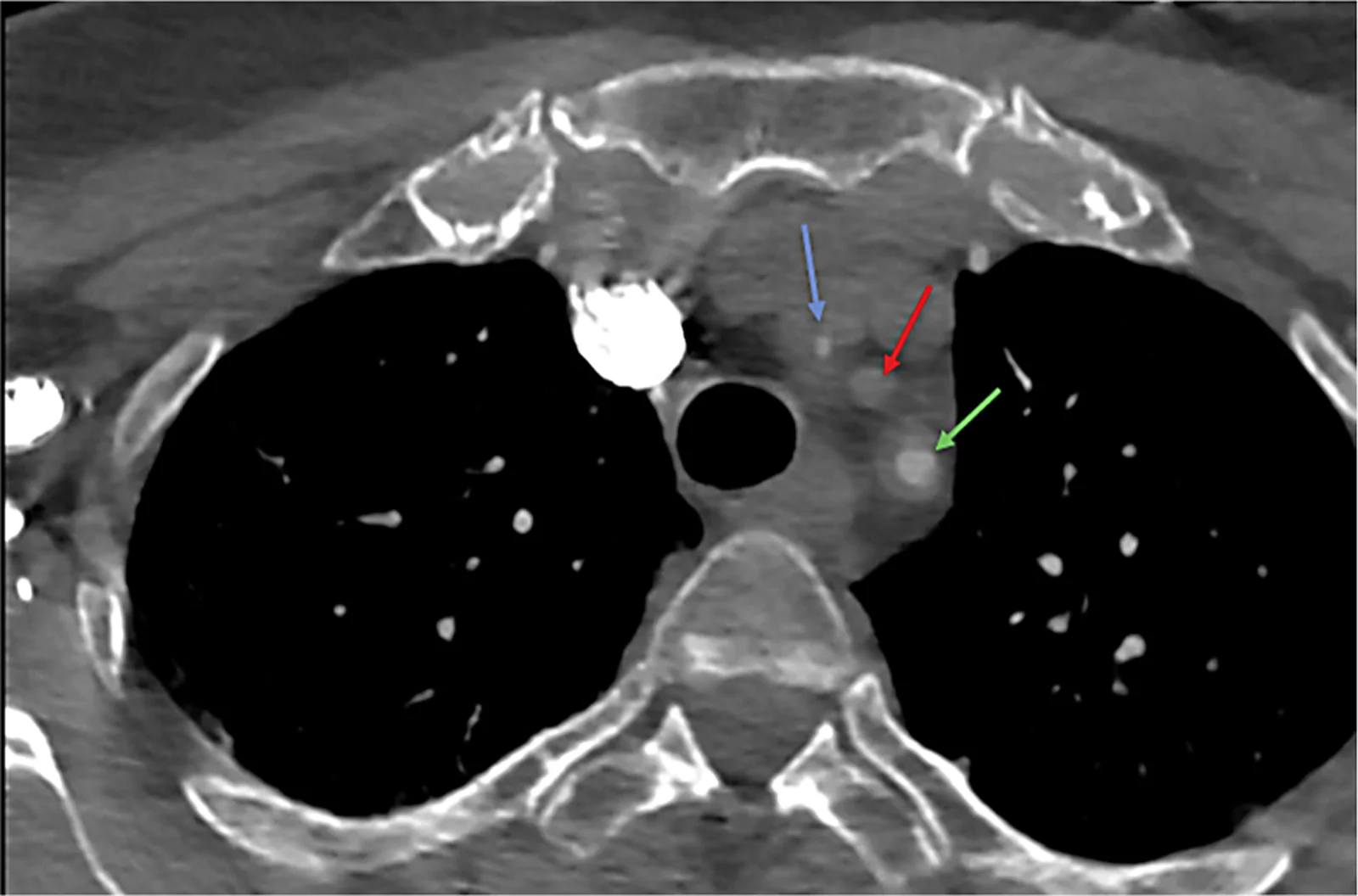
Axial CTA of the supra-aortic trunks demonstrating concentric wall thickening and severe luminal narrowing of the right brachiocephalic trunk (blue arrow), left subclavian artery (green arrow), and left common carotid artery (red arrow), without definite intraluminal filling defects.

**Fig. 3.**
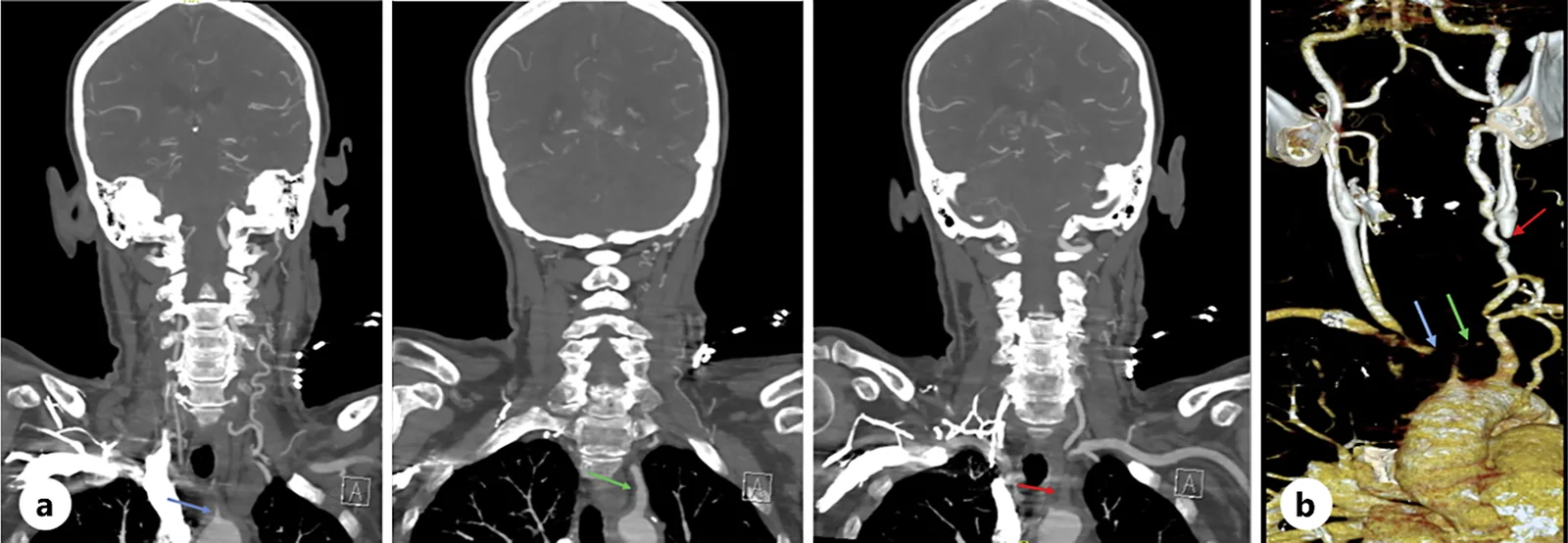
**a** Coronal CTA demonstrating the longitudinal extent of the lesions of the right brachiocephalic trunk (blue arrow), left subclavian artery (green arrow), and left common carotid artery (red arrow). **b** CTA 3D reconstruction of the supra-aortic trunks demonstrating the longitudinal extent of the lesions of the right brachiocephalic trunk (blue arrow), left subclavian artery (green arrow), and left common carotid artery with distal reconstitution beyond the carotid bifurcation (red arrow).

Brain magnetic resonance imaging confirmed subacute infarcts in the right anterior and middle cerebral artery territories, as well as older infarcts in the right frontoparietal and temporo-occipital regions (shown in Fig. 4). Although the multifocal distribution initially raised concern for an embolic mechanism, the overall pattern, together with the extensive proximal arterial disease, was ultimately considered more consistent with a hemodynamic (watershed) mechanism.

**Fig. 4.**
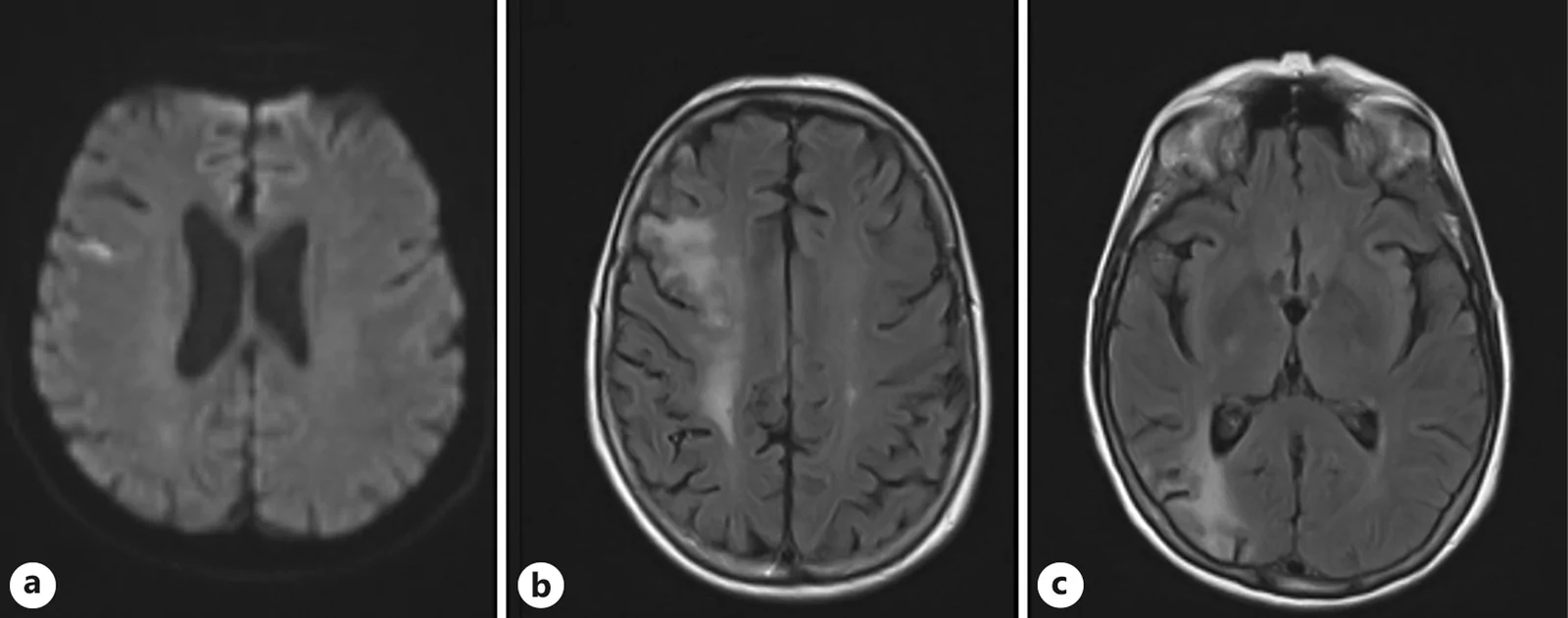
**a** Diffusion-weighted brain MRI demonstrating a recent infarct in the right frontal lobe. **b**, **c** T2-FLAIR brain MRI showing extensive chronic ischemic changes, predominantly affecting the right hemisphere. MRI, magnetic resonance imaging.

Transthoracic echocardiography showed normal cardiac function without significant valvular disease. Transesophageal echocardiography revealed circumferential thickening of the aortic arch and descending aorta, a normal left atrial appendage, and a non-patent foramen ovale. Continuous cardiac monitoring did not reveal any cardioembolic source, including atrial fibrillation or other significant arrhythmias.

Taken together, these findings did not identify a cardioembolic source. After integration of all available imaging modalities, the vascular abnormalities were therefore interpreted as most consistent with diffuse arterial wall thickening rather than thrombus formation. In this context, intravenous heparin was discontinued.

Routine thrombophilia screening, including antiphospholipid antibodies, was normal. Autoimmune serologic screening (including rheumatoid factor, antinuclear antibodies, and antineutrophil cytoplasmic antibodies) and extended viral, bacterial, and parasitic investigations were negative.

Whole-body ^18^F-fluorodeoxyglucose positron emission tomography-CT showed no signs of malignancy and confirmed irregular wall thickening of the aortic arch, descending aorta, right brachiocephalic trunk (severely stenotic), and left common carotid artery (occluded at its origin), without significant FDG uptake (shown in Fig. 5). Additional stenoses were noted in the iliac arteries, celiac trunk, and origin of the superior mesenteric artery, confirmed by abdominal CTA, which also showed a left renal infarct.

**Fig. 5.**
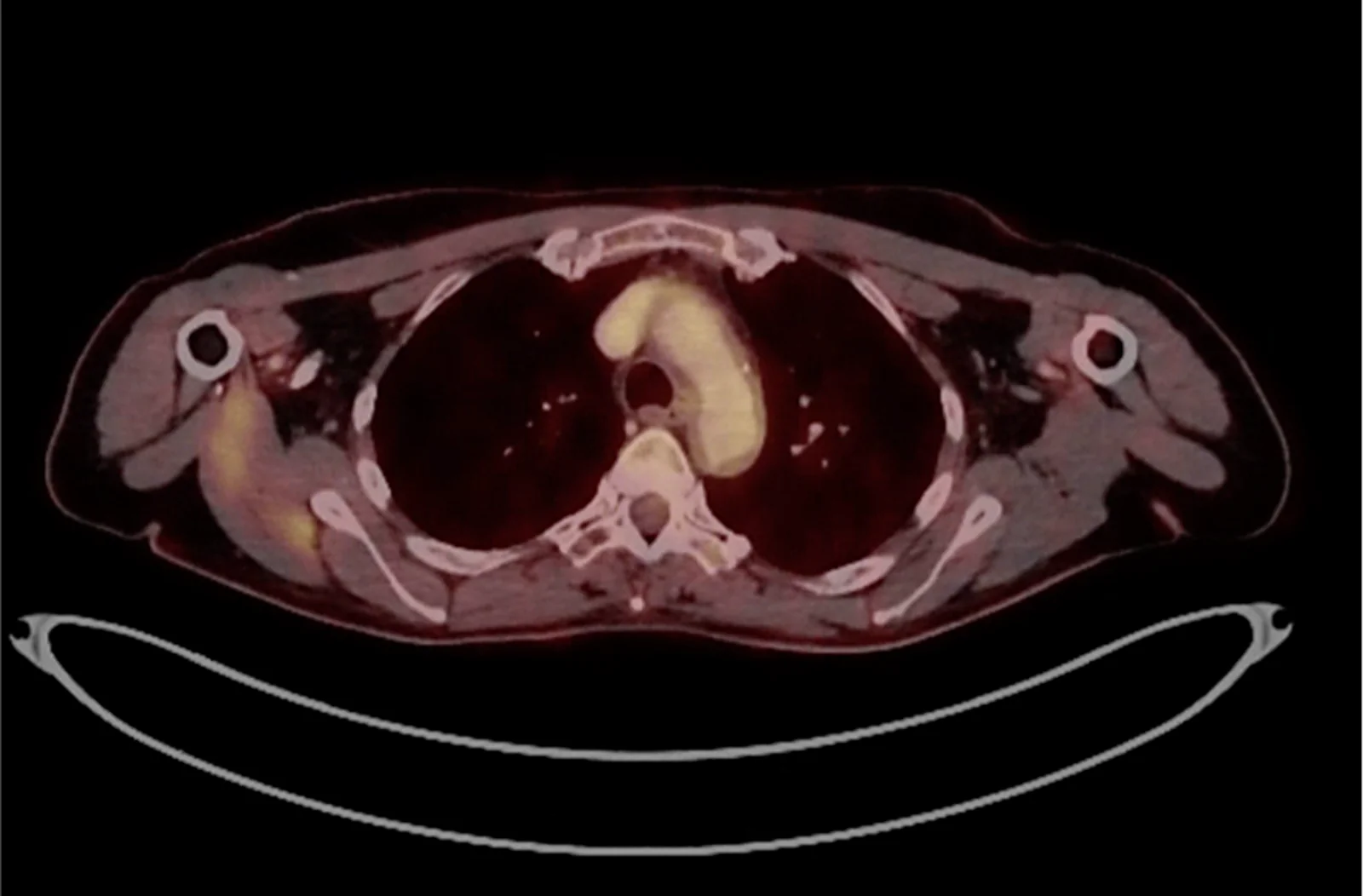
Whole-body FDG PET-CT showing irregular circumferential thickening of the aortic arch walls, without significant FDG uptake. FDG PET-CT, ^18^F-fluorodeoxyglucose positron emission tomography-computed tomography.

Bilateral temporal artery ultrasound was normal. Temporal artery biopsy, performed within 2 weeks of corticosteroid initiation, revealed fragmentation of the internal elastic lamina involving approximately half of the arterial circumference, with sparse inflammatory infiltrates. These findings were interpreted as cicatricial changes consistent with healed arteritis. Genetic testing was negative for HLA-B*52 and HLA-DR4.

## Therapy

Considering the high index of suspicion for LVV, she was started on high-dose intravenous methylprednisolone (500 mg for 3 days) followed by oral administration (48 mg daily). She was started on weekly methotrexate and underwent brachiocephalic trunk and right subclavian stenting with right internal carotid artery balloon dilation about 6 weeks from therapy initiation in order to mitigate any flare-up risk.

## Outcome and Follow-Up

Following a 4-month hospitalization in our stroke and rehabilitation unit, the patient’s motor deficits improved but remained disabling in daily life. She was able to ambulate with a walker under supervision and continued to experience impaired fine motor function in the left hand. Neuropsychological assessment revealed persistent cognitive impairment. Considering these findings, alongside the patient’s personal and familial preferences and a multidisciplinary team discussion, institutional placement was deemed the most appropriate disposition.

## Discussion

This case highlights the substantial diagnostic challenges posed by LVV in elderly patients presenting with ischemic stroke. Stroke is an uncommon manifestation of systemic vasculitis, particularly as an initial presentation in the absence of systemic inflammatory signs. Given the predominance of atherosclerotic and cardioembolic etiologies in this age group, vasculitis is rarely considered early in the diagnostic process.

The differential diagnosis in this case includes several noninflammatory large-vessel pathologies. Aortic atherosclerosis may present with luminal irregularities and plaque formation, often associated with focal, eccentric wall thickening and calcified plaques, in contrast to the diffuse, concentric, and relatively symmetric wall thickening observed in this patient. In addition, there was no imaging evidence of significant calcified or ulcerated atherosclerotic plaque burden. In this patient, the initial challenge was distinguishing periaortitis from intramural aortic thrombus on CTA and transesophageal echocardiography. Circumferential aortic wall thickening may mimic mural thrombus, especially in acute ischemic stroke. Similar diagnostic pitfalls have been previously reported in the literature, underscoring the limitations of conventional vascular imaging in differentiating inflammatory from thrombotic aortic pathology [5]. The extent of the lesion as well as the absence of a distinct intraluminal filling defect made the diagnosis of mural thrombus less likely. Aortic dissection was also unlikely given the absence of chest pain and of an intimal flap or double lumen on multimodal imaging. Connective tissue disorders and other noninflammatory vasculopathies such as Moyamoya disease and fibromuscular dysplasia were also considered but were not supported by the clinical, biological, or imaging findings. The extensive, multi-territorial, and relatively symmetric involvement of large arteries ultimately favored an inflammatory vasculopathy.

GCA typically affects individuals over 50 years of age and classically presents with cranial symptoms such as new-onset headache, jaw claudication, scalp tenderness, or visual disturbances. Constitutional symptoms, including fatigue, fever, morning stiffness, and proximal girdle pain, are also common. Stroke is an uncommon manifestation of GCA, occurring in approximately 2.8% of cases, and is more frequently associated with extracranial large-vessel involvement.

However, atypical presentations of GCA are increasingly recognized. Nearly 40% of patients exhibit at least one atypical feature, and approximately 18% present exclusively with atypical manifestations. In particular, GCA without cranial involvement has emerged as a distinct phenotype, often characterized by normal or minimally elevated inflammatory markers and predominant involvement of the aorta and its major branches [6].

In response to these diagnostic challenges, the 2022 ACR/EULAR classification criteria for GCA incorporate laboratory, imaging, and histopathological findings, in addition to clinical features [7]. Nevertheless, important limitations remain, especially in patients with exclusive extracranial disease. In a case series by de Boysson et al. [6], 35% of patients presenting solely with extracranial manifestations did not fulfill the ACR classification criteria, highlighting reduced sensitivity of these criteria in this subgroup. Furthermore, the diagnostic yield of temporal artery biopsy in large-vessel-predominant GCA remains controversial [8].

Despite extensive evaluation, our patient did not meet the 2022 ACR/EULAR criteria for GCA. She lacked cranial symptoms, had normal inflammatory markers, and showed no hypermetabolic vascular uptake on 18 F-fluorodeoxyglucose positron emission tomography-CT. It is important to note that LVV may occur with absent FDG uptake [9] and normal inflammatory markers [10], particularly in chronic or partially treated disease, which may further complicate diagnosis. Temporal artery biopsy revealed only cicatricial changes without evidence of active inflammation. Even if considered positive, the biopsy would yield a total score of five, below the threshold for classification. Taken together, these findings precluded a definitive diagnosis of GCA according to current criteria.

Conversely, the patient fulfilled multiple criteria consistent with TAK according to the 2022 ACR/EULAR classification [9]. These included female sex, the presence of vascular bruits, absent upper extremity pulse, significant systolic blood pressure discrepancy between arms, involvement of the carotid and brachiocephalic arteries, symmetrical disease of paired vessels, and extensive involvement of the abdominal aorta and its major branches. However, TAK requires an absolute age criterion of ≤60 years at diagnosis, which our patient marginally exceeded. While the age criterion was mandatory in the original Ishikawa diagnostic criteria, it was later made nonmandatory in the 1990 ACR diagnostic criteria. This criterion remains controversial, as strict adherence may result in misclassifying late-onset TAK as GCA, even when the vascular phenotype is more adequate for TAK [10].

Genetic markers can provide additional support in ambiguous cases. HLA-B*52 and HLA-DR4 have been associated with TAK [11] and GCA [12], respectively, although neither is included in current classification criteria. In our patient, the absence of these alleles did not contribute to the diagnostic evaluation.

These diagnostic uncertainties directly affect long-term management. The patient was treated with systemic corticosteroids and methotrexate as a steroid-sparing agent, consistent with standard LVV management. However, had a definitive diagnosis of GCA been established, anti-interleukin-6 receptor therapy with tocilizumab could have been considered, given its proven efficacy and its steroid-sparing benefits [13]. Beyond therapeutic aspects, disease progression differs between TAK and GCA, highlighting the need for tailored follow-up.

Although clear epidemiological and geographical distinctions exist between TAK and GCA, the underlying pathophysiology remains poorly understood. Our patient appears to fall along the historically described TAK-GCA spectrum, encompassing features of late-onset TAK and GCA with primarily large-vessel involvement, both of which are insufficiently accounted for in current diagnostic criteria [14].

For stroke physicians, this case highlights several red flags that should prompt early consideration of LVV in atypical stroke presentations, even in the absence of elevated inflammatory markers. These include marked inter-arm blood pressure discrepancies, absent peripheral pulses, diffuse arterial bruits, and imaging findings of extensive supra- and infra-aortic involvement, with diffuse, concentric vessel wall thickening. Early recognition is essential, as management differs fundamentally from that of atherosclerotic or cardioembolic stroke mechanisms. Delayed diagnosis may lead to inappropriate treatment strategies and postponement of immunosuppressive therapy.

## Conclusion

Overall, this case illustrates the limitations of existing classification frameworks when confronted with borderline or overlapping LVV phenotypes, particularly in elderly patients with atypical presentations. It emphasizes the need for a pragmatic, multidisciplinary approach that integrates clinical judgment with imaging and histopathological findings, rather than rigid adherence to age-based or biomarker-dependent criteria.

## Statement of Ethics

No supplementary interventions have been performed other than the indicated diagnostic interventions. Ethical approval is not required for this study in accordance with local, national, and institutional guidelines. Written informed consent was obtained from the patient for publication of this case report and any accompanying images.

## Conflict of Interest Statement

The authors have no conflicts of interest to declare.

## Funding Sources

This study was not supported by any sponsor or funder.

## Author Contributions

Rami Abdul Malak is the first author, who has written the article and was involved in the management of the patient. Idriss Youcef Zoubeïdi, Julien Vanderhulst, and Anissa Ourtani critically reviewed the article and were involved in the management of the patient.

## Data Availability

The data supporting the findings of this study are included within the article. No additional datasets were generated or analyzed. Further individual patient data are not publicly available due to privacy and ethical restrictions but may be considered upon reasonable request and with the appropriate institutional approval of Brugmann University Hospital, Belgium. Further inquiries can be directed to the corresponding author.
